# Simulating tDCS electrode placement to stimulate both M1 and SMA enhances motor performance and modulates cortical excitability depending on current flow direction

**DOI:** 10.3389/fnins.2024.1362607

**Published:** 2024-07-01

**Authors:** Takatsugu Sato, Natsuki Katagiri, Saki Suganuma, Ilkka Laakso, Shigeo Tanabe, Rieko Osu, Satoshi Tanaka, Tomofumi Yamaguchi

**Affiliations:** ^1^Department of Physical Therapy, Yamagata Prefectural University of Health Sciences, Yamagata, Japan; ^2^Department of Rehabilitation Medicine, Tokyo Bay Rehabilitation Hospital, Narashino, Japan; ^3^Graduate School of Health Sciences, Yamagata Prefectural University of Health Sciences, Yamagata, Japan; ^4^Department of Electrical Engineering and Automation, Aalto University, Espoo, Finland; ^5^Faculty of Rehabilitation, School of Health Sciences, Fujita Health University, Toyoake, Japan; ^6^Faculty of Human Sciences, Waseda University, Tokorozawa, Japan; ^7^Laboratory of Psychology, Hamamatsu University School of Medicine, Hamamatsu, Japan; ^8^Department of Physical Therapy, Faculty of Health Science, Juntendo University, Tokyo, Japan; ^9^Department of Physical Therapy, Human Health Sciences, Graduate School of Medicine, Kyoto University, Kyoto, Japan

**Keywords:** primary motor cortex, supplementary motor area, non-invasive brain stimulation, lower limb, muscle strength, rehabilitation

## Abstract

**Introduction:**

The conventional method of placing transcranial direct current stimulation (tDCS) electrodes is just above the target brain area. However, this strategy for electrode placement often fails to improve motor function and modulate cortical excitability. We investigated the effects of optimized electrode placement to induce maximum electrical fields in the leg regions of both M1 and SMA, estimated by electric field simulations in the T1and T2-weighted MRI-based anatomical models, on motor performance and cortical excitability in healthy individuals.

**Methods:**

A total of 36 healthy volunteers participated in this randomized, triple-blind, sham-controlled experiment. They were stratified by sex and were randomly assigned to one of three groups according to the stimulation paradigm, including tDCS with (1) anodal and cathodal electrodes positioned over FCz and POz, respectively, (A-P tDCS), (2) anodal and cathodal electrodes positioned over POz and FCz, respectively, (P-A tDCS), and (3) sham tDCS. The sit-to-stand training following tDCS (2 mA, 10 min) was conducted every 3 or 4 days over 3 weeks (5 sessions total).

**Results:**

Compared to sham tDCS, A-P tDCS led to significant increases in the number of sit-to-stands after 3 weeks training, whereas P-A tDCS significantly increased knee flexor peak torques after 3 weeks training, and decreased short-interval intracortical inhibition (SICI) immediately after the first session of training and maintained it post-training.

**Discussion:**

These results suggest that optimized electrode placement of the maximal EF estimated by electric field simulation enhances motor performance and modulates cortical excitability depending on the direction of current flow.

## 1 Introduction

Transcranial direct current stimulation (tDCS) is a non-invasive cortical stimulation procedure in which weak direct currents polarize target brain regions (Nitsche and Paulus, 2000). The application of tDCS to motor-related cortical areas transiently alters cortical excitability and improves motor performance in healthy individuals and patients with stroke (Jeffery et al., 2007; Tanaka et al., 2009, 2011; Madhavan et al., 2011; Tatemoto et al., 2013; Sriraman et al., 2014; Chang et al., 2015; Vitor-Costa et al., 2015; Angius et al., 2016, 2018; Montenegro et al., 2016; Washabaugh et al., 2016). However, the conventional method of placing tDCS electrodes just above the target brain area often fails to modulate excitability within the target cortex or improve motor performance, frequently limited by significant inter-individual variability (López-Alonso et al., 2014, 2015; Wiethoff et al., 2014; Chew et al., 2015; Yamaguchi et al., 2016; Maeda et al., 2017).

One possible source of this inter-individual variability is the variability of tDCS-generated electrical fields (EFs) (Laakso et al., 2019). The EFs in the brain depend on the electrical resistance of the tissues, i.e., scalp, skull, meninges, and cerebrospinal fluid (CSF), between the electrode and the brain (Truong et al., 2013; Laakso et al., 2015; Opitz et al., 2015). To reduce inter-individual variability in tDCS-induced effects, Laakso et al. (2015, 2016) proposed a systematic way to estimate EFs induced by tDCS at the population level by registering calculated EFs with structural brain magnetic resonance imaging (MRI). Electric field simulation provides information that is useful for optimizing tDCS settings (intensity, electrode size, electrode placement), which in turn may generate EFs with minimized variation among individuals (Evans et al., 2020; Mikkonen et al., 2020). Previous studies speculated that motor-evoked potential (MEP) and cerebral blood flow changes induced by tDCS were related to EF values in the target brain area (Mosayebi-Samani et al., 2021). However, conventional tDCS electrode placement in healthy controls and stroke patients often fails to induce sufficient EFs to achieve the desired effects within the targeted area (van der Cruijsen et al., 2022; Yoon et al., 2024), making the use of electrical field simulations to optimize electrode placement a novel approach for modulating cortical excitability and enhancing motor performance in stroke patients (van der Cruijsen et al., 2022).

Another possible factor of variability is the tDCS-induced current flow. One study reported that tDCS over primary motor cortex (M1) with posterior to anterior (P-A) current flow decreased corticospinal excitability (Rawji et al., 2018). Another group studying motor task learning under tDCS over M1 reported that using anterior to posterior (A-P) current flow disturbed the retention of learned skills (Hannah et al., 2019). These studies suggest that the tDCS-induced current flow plays an important role in mediating changes in corticospinal excitability and motor learning. However, to the best of our knowledge, the effects of current flow direction on motor performance and cortical excitability under tDCS using optimized electrode placement have not been investigated. New insights obtained by such investigation may help enhance tDCS effects, which in turn could benefit patients with neurological disorders in neurorehabilitation with increased cortical excitability and improve motor performance.

The sit-to-stand movement, a typical daily activity (Alexander et al., 2000), relies on the lower limb fields of M1 for essential motor signaling to induce muscle contraction during this action (Pearson, 2000). The supplementary motor area (SMA) is integral for the coordination and execution of motor programs, particularly in skilled movements and postural control (Mihara et al., 2008, 2012; Fujimoto et al., 2014). Considering this, we selected FCz and POz electrode placements in our study to induce the maximal average EFs in M1 and SMA on both sides, as revealed by electric field simulations. These simulations demonstrated that conventional electrode placements above the target brain area do not achieve maximal EFs in these regions (Table 1). Moreover, the extent of EFs induced in the target area correlates with the cortical excitability changes induced by tDCS (Mosayebi-Samani et al., 2021), leading to our decision not to include a conventional tDCS group in this study.

**Table 1:** Electric field simulation of each electrode montage.

		Electric field strength (V/m)				
		M1		SMA		
Electrode 1	Electrode 2	Left	Right	Left	Right	Average
Fz	Extracephalic	0.29 ± 0.04	0.29 ± 0.04	0.43 ± 0.07	0.43 ± 0.08	0.36 ± 0.05
FCz	Extracephalic	0.40 ± 0.06	0.40 ± 0.06	0.58 ± 0.10	0.57 ± 0.11	0.49 ± 0.07
Cz	Extracephalic	0.50 ± 0.07	0.51 ± 0.08	0.63 ± 0.11	0.63 ± 0.11	0.57 ± 0.09
CPz	Extracephalic	0.54 ± 0.09	0.55 ± 0.09	0.52 ± 0.09	0.52 ± 0.09	0.53 ± 0.08
Pz	Extracephalic	0.50 ± 0.09	0.51 ± 0.08	0.37 ± 0.06	0.38 ± 0.06	0.44 ± 0.07
Fz	Iz	0.41 ± 0.05	0.42 ± 0.05	0.52 ± 0.08	0.52 ± 0.09	0.47 ± 0.06
FCz	Iz	0.50 ± 0.06	0.51 ± 0.07	0.62 ± 0.10	0.62 ± 0.11	0.56 ± 0.08
Cz	Iz	0.58 ± 0.07	0.58 ± 0.08	0.61 ± 0.11	0.61 ± 0.11	0.60 ± 0.08
CPz	Iz	0.55 ± 0.09	0.56 ± 0.09	0.45 ± 0.08	0.46 ± 0.08	0.50 ± 0.08
Pz	Iz	0.43 ± 0.08	0.44 ± 0.07	0.28 ± 0.05	0.28 ± 0.05	0.36 ± 0.06
Fz	Fpz	0.11 ± 0.02	0.12 ± 0.03	0.22 ± 0.05	0.22 ± 0.06	0.17 ± 0.03
FCz	Fpz	0.24 ± 0.05	0.24 ± 0.05	0.46 ± 0.10	0.46 ± 0.10	0.35 ± 0.07
Cz	Fpz	0.39 ± 0.07	0.40 ± 0.08	0.65 ± 0.12	0.65 ± 0.11	0.52 ± 0.09
CPz	Fpz	0.51 ± 0.09	0.53 ± 0.10	0.65 ± 0.10	0.65 ± 0.10	0.59 ± 0.09
Pz	Fpz	0.56 ± 0.09	0.58 ± 0.08	0.56 ± 0.08	0.56 ± 0.08	0.56 ± 0.07
POz	Fpz	0.51 ± 0.07	0.52 ± 0.06	0.47 ± 0.06	0.47 ± 0.06	0.49 ± 0.05
Fz	POz	0.58 ± 0.07	0.59 ± 0.07	0.63 ± 0.09	0.62 ± 0.10	0.61 ± 0.07
FCz	POz	0.63 ± 0.07	0.64 ± 0.07	0.66 ± 0.11	0.66 ± 0.11	0.65 ± 0.08
Cz	POz	0.62 ± 0.08	0.62 ± 0.08	0.55 ± 0.10	0.55 ± 0.10	0.59 ± 0.08
CPz	POz	0.44 ± 0.07	0.44 ± 0.07	0.30 ± 0.06	0.30 ± 0.06	0.37 ± 0.06
Fz	Pz	0.59 ± 0.08	0.61 ± 0.08	0.68 ± 0.10	0.68 ± 0.11	0.64 ± 0.08
FCz	Pz	0.60 ± 0.08	0.61 ± 0.08	0.67 ± 0.11	0.67 ± 0.12	0.63 ± 0.08
Cz	Pz	0.49 ± 0.07	0.50 ± 0.06	0.45 ± 0.09	0.45 ± 0.09	0.48 ± 0.07

Data are represented as mean ± standard deviation over 62 head models.

We hypothesized that the jointly optimal tDCS for SMA and M1, as estimated by electric field simulation, would positively influence motor performance in the sit-to-stand movement, as assessed by muscle strength and neurophysiological assessments; MEPs and short-inter-cortical inhibition (SICI). To address this hypothesis, we used electric field simulation to determine the electrode placement that maximized EFs in SMA and M1 and then examined how tDCS influenced sit-to-stand performance, muscle strength, and cortical excitability in healthy individuals.

## 2 Materials and methods

### 2.1 Participants

The study involved 36 healthy young college student volunteers (18 women; aged 21 ± 1 year) (Table 2). Out of 37 initial participants, one was excluded due to medication affecting the central nervous system. The sample size, set at 12 per group, was determined by a power analysis referencing Tanaka et al. (2009), and aligns with the recommended minimum for pilot studies (Julious, 2005).

**Table 2:** Participant characteristics.

	A-P tDCS group (*n* = 12)	P-A tDCS group (*n* = 12)	Sham tDCS group (*n* = 12)	*p*-value
Age (years)	21 (1)	21 (1)	22 (1)	0.09
Sex, male/female (number)	6/6	6/6	6/6	–
Height (cm)	164.6 (9.8)	166.3 (8.2)	163.5 (6.4)	0.72
Weight (kg)	56.5 (8.2)	62.9 (13.5)	56.9 (6.5)	0.68

Data are presented as the mean (standard deviation). After confirmation of the normality of each dataset, one-way analysis of variance was utilized for normally distributed data, and Kruskal–Wallis test was employed for non-normally distributed data. To specify the effect of group difference, a *p*-value was conducted. tDCS, transcranial direct current stimulation; A-P tDCS, anterior-to-posterior current flow tDCS; P-A tDCS, posterior-to-anterior current flow tDCS.

Participants had no history of orthopedic or neurological diseases and were not treated with medications that would affect the central nervous system. To control factors that could influence the effectiveness of non-invasive brain stimulation, participants were asked to avoid vigorous physical activity and consumption of alcohol and caffeine during the experiment period and to ensure adequate sleep the night before the experiment to avoid sleep deprivation (Guerra et al., 2020).

All participants gave written informed consent before participating in the experiment. This study was approved by the Ethics Committee of Yamagata Prefectural University of Health Sciences (approval number: 1806–06) and was performed according to the ethical standards of the Declaration of Helsinki.

### 2.2 Experimental procedure

The study was a randomized, triple-blind, sham-controlled trial. Intervention conditions were concealed from participants, outcome assessors, and data analysts. Participants were stratified by sex and randomly assigned to groups using float numbers between 0 and 1 from a continuous uniform distribution. Assignment was based on whether the number drawn is smaller or equal in value compared to 0.5 or larger than 0.5. These numbers were generated by a third party unrelated to evaluation or intervention (Microsoft, Washington, United States).

**Figure 1:**
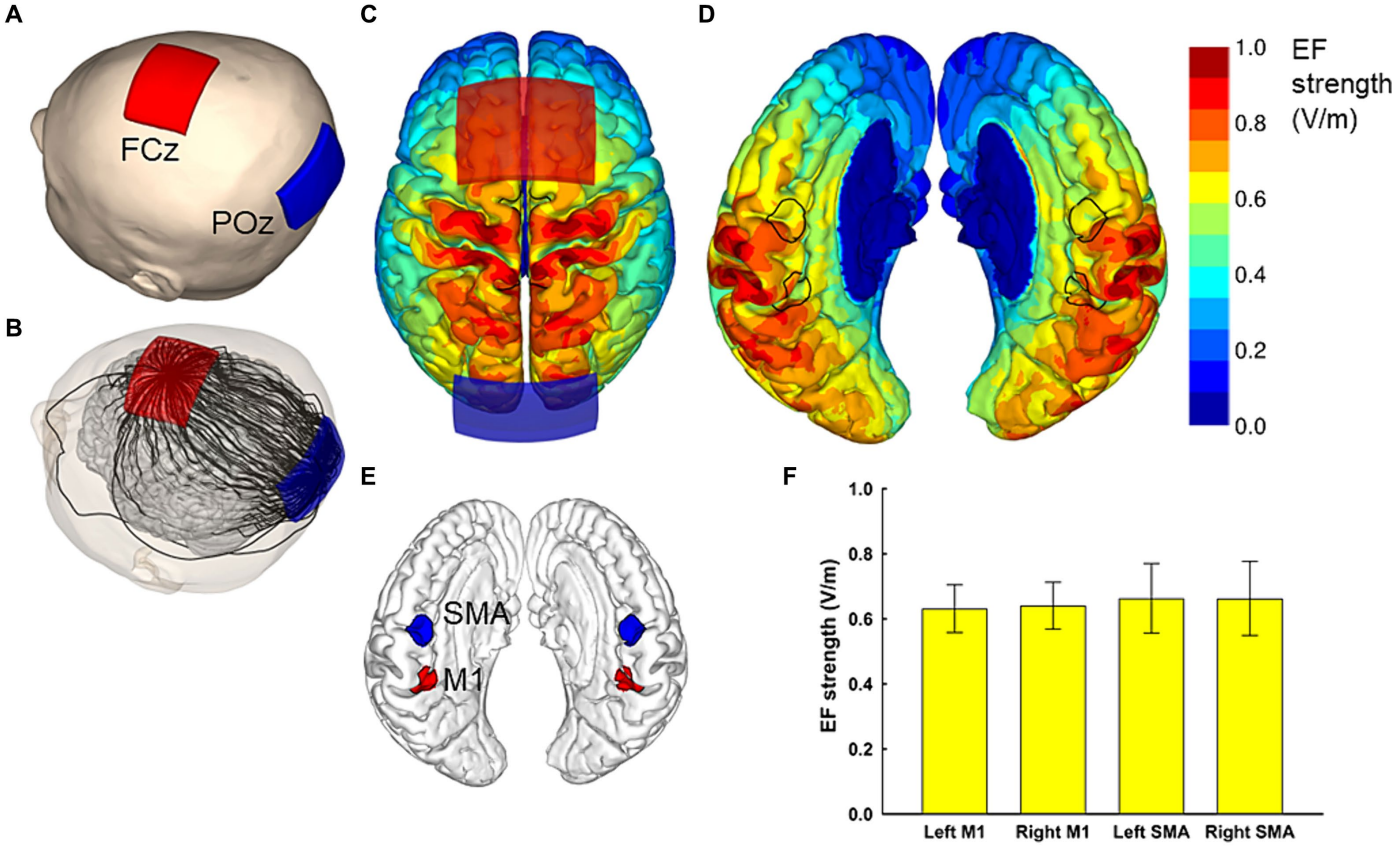
Electric field simulations. The optimal electrode locations were at FCz and POz **(A)**, which produced a current flow in the anterior–posterior or posterior–anterior direction, depending on the polarity of the electrodes. A streamline plot visualizes the current direction between the electrodes in a representative head model **(B)**. The Electric field (EF) strengths averaged over 62 head models and registered to a common template brain are shown from the superior direction at a depth of 1 mm below the surface of the grey matter **(C,D)**. In **(D)**, the hemispheres have been separated to visualize the EF strength along the interhemispheric fissure, with the black outlines showing the regions of interest (ROI) that correspond to the left and right SMA and M1 **(E)**. The average EF strengths over each ROI are presented as mean ± standard deviation **(F)**.

**Figure 2:**
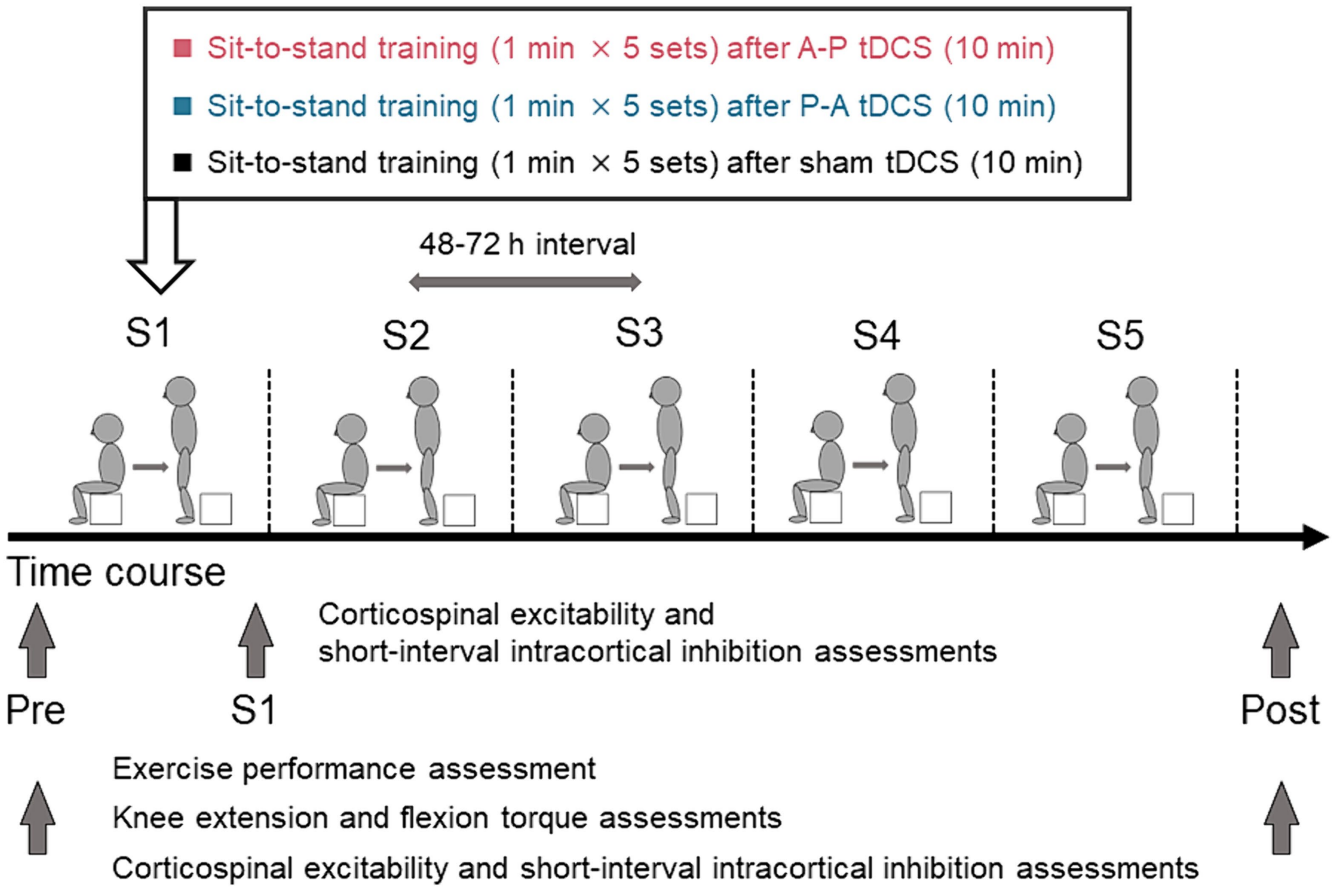
Schematics and timeline of the experimental procedures. Healthy participants were randomly assigned to one of three groups in which sit-to-stand training followed tDCS using different current flow paradigms: (i) anterior–posterior (A-P tDCS), (ii) posterior–anterior (P-A tDCS), and (iii) no current (sham tDCS). A single training session consisted of 5 sets of 1 min sit-to-stand with a 180-s rest period between sets. The training was conducted in 5 sessions over 3 weeks, with 48 to 72 h between sessions. Sit-to-stand were assessed before tDCS intervention (Pre), at each of the five sessions (S1, S2, S3, S4, S5), and after training sessions (Post). The single- and paired-pulse TMS were assessed at Pre, S1, and Post and knee extensor and flexor peak torques were assessed at Pre and Post.

To induce the maximal EFs in both SMA and M1, the optimal electrode configuration was determined through computer simulations (see “Electric field simulation”). These simulations indicated that the field maximizing electrode-pair positions were at FCz and POz (Figure 1). Participants received tDCS with one of the following three different electrode placements: (1) anodal and cathodal electrodes positioned over FCz and POz, respectively, resulting in anterior-to-posterior current flow (A-P tDCS); (2) the reverse arrangement, where anodal and cathodal electrodes were positioned over POz and FCz, respectively, resulting in the reverse (posterior to anterior) currents (P-A tDCS), and (3) no current passed (sham tDCS) (Figure 2).

Participants were stratified by sex and were randomly assigned to one of the above three groups. All participants underwent the sit-to-stand training following tDCS intervention. The sit-to-stand training was conducted once every 3 or 4 days for 3 weeks (a total of 5 sessions). Exercise performance, measured by the number of sit-to-stands executed in a minute, was assessed pre-training, during each training session, and post-training. Muscle strength (knee extension and flexion peak torques) was measured pre- and post-training. Cortical excitability was measured pre-training, immediately after the first training session, and post-training. Pre-training assessments were conducted between 48 and 72 h before the first training session. Post-training assessments were conducted between 48 and 72 h after the final training session.

### 2.3 tDCS setting

tDCS was administered using a DC-Stimulator-Plus (neuroConn, GmbH, Germany) connected to a pair of sponge surface electrodes, each with a surface area of 35 cm^2^, soaked in a 0.9% NaCl saline solution. With the electrodes placed on the scalp of the participant, a direct current of 2 mA was applied for 10 min. Skin pre-treatment agents and alcohol swabs were used to reduce scalp skin resistance at the electrode contact area. The electrode placement was consistently maintained at the same positions, determined by measuring the length of the participant’s head with a tape measure during each session. For the sham condition, the same procedure was performed; however, the current was turned off after the first 15 s to mimic the transient skin sensation felt at the beginning of the direct current. Intervention condition was masked to participants, outcome assessors, and data analysts.

### 2.4 Electric field simulation

In order to induce the maximal EF strength in both SMA and M1, the optimal electrode montage was determined based on computer simulation of the EFs, similarly to our previous studies (Laakso et al., 2016; Fujimoto et al., 2017).

For the computer simulations of the EF, we utilized 62 individual MRI-based anatomical models, consistent with those used in our previous studies (Laakso et al., 2016; Fujimoto et al., 2017). Each model, with an isotropic resolution of 0.5 mm, was constructed from the segmentation of T1- and T2-weighted (T1w and T2w) MRI data. FreeSurfer image analysis software was employed to segment the brain, including gray and white matter (Dale et al., 1999; Fischl et al., 1999; Fischl and Dale, 2000). Other tissue compartments were segmented as follows: The inner and outer boundaries of the skull, along with the outer surface of the scalp, were identified from the MRI data. An experienced investigator utilized semi-automatic image processing techniques, including thresholding, opening/closing, smoothing, and region growing on the T1w and T2w data, with the parameter values chosen on a per-subject basis. The spaces between the brain, inner skull surface, outer skull surface, and the scalp surface were automatically segmented into multiple tissue types using both MRI data and geometrical operations. The space between the skull and brain was segmented into CSF (large T2w), blood (small T2w), and dura (outer 1.5 mm which is not CSF or a large dural vein). The space between the inner and outer boundaries of the skull was segmented into compact bone (small T2w but having at least a 1-mm-thick inner layer and 1.5-mm-thick outer layer) and spongy bone (large T2w). Lastly, the space between the outer boundary of the skull and the surface of the scalp was stratified into fat (large T1w), muscle (small T1w), and skin (small T1w, at least 2-mm thick, at most 1-cm thick including subcutaneous fat) The quality of each model was verified by two independent examiners (Laakso et al., 2016). The electrical conductivities assigned to each tissue were (Laakso et al., 2016): gray matter (0.2 S/m), white matter (0.14 S/m), blood (0.7 S/m), compact bone (0.008 S/m), spongy bone (0.027 S/m), dura (0.16 S/m), CSF (1.8 S/m), muscle (0.16 S/m), skin and fat (0.08 S/m), and eye (1.5 S/m). The sensitivity of the EF to conductivity values was assessed by varying bone conductivity by ±50% and the CSF conductivity by ±10% and repeating all calculations.

The EF was determined as the gradient of the electric scalar potential (ϕ) , which was obtained by numerically solving ∇.σ∇ϕ=i with homogeneous Neumann boundary conditions, where σ is the conductivity (S/m) and i is the current source/sink (A/m^3^). The finite element method (FEM) with 0.5 mm cubical first-order elements was employed for numerical solution (Laakso and Hirata, 2012; available at https://version.aalto.fi/gitlab/ilaakso/vgm-fem). The stimulation electrodes were modeled identically to our previous studies (Laakso et al., 2016; Fujimoto et al., 2017), as a 1 mm thick rubber sheet embedded in a saline-soaked sponge (1.6 S/m), with size, shape, and current intensity identical to those in the actual experiment (Figure 1). After obtaining the EF, it was interpolated to a polygonal surface reconstruction of the brain at 1 mm depth below the gray matter surface. As detailed in Laakso et al. (2016), the individual surface EFs were then registered with each other and mapped to the Montreal Neurological Institute (MNI) ICBM 2009a nonlinear asymmetric template (Fonov et al., 2009, 2011) using FreeSurfer and the spherical demons algorithm (Yeo et al., 2010). This process enabled determination of the population-average EFs of the bilateral SMA and M1 in the MNI space.

For the optimization of the electrode montage, we considered four regions of interest. The regions of interest for M1 and SMA had a radius of 1 cm and were centered at [± 9, − 39, 54] and [± 3, − 9, 60] in the standard brain space, respectively. Our aim was to find a montage of two electrodes that produces symmetric bilateral stimulation and maximizes the average EF strength over the four regions of interest. To achieve symmetric stimulation, both the anode and cathode needed to be placed on the midline of the head. For practical applicability, the electrode locations were selected using the International 10–10 system: The first electrode was placed at Fz, FCz, Cz, CPz, or Pz, and the second electrode at an extracephalic location, frontally (Fpz), posteriorly (Iz), or close to the stimulated areas (POz or Pz). The electrodes were oriented so that the long edges of the electrodes were perpendicular to the posterior–anterior direction. Any locations which would have caused the electrodes to overlap were excluded, leaving 23 electrode montages, listed in the first two columns of Table 1.

The EF produced by each montage were calculated for all 62 head models, and the EF strength averaged over each/all regions of interest was determined. Finally, the mean and standard deviation of these average values were calculated over the 62 head models. The results for each studied electrode montage are provided in Table 1. It was found that the FCz-POz configuration induced the largest mean value of the average EF strength over the four regions of interests, closely followed by the Fz-Pz montage. At the individual level, FCz-POz was the optimal montage in 32/62 models and Fz-Pz in 22/62 models (Supplementary Table S1). Repeating the EF analyses for altered bone and CSF conductivities indicated that either the FCz-POz or the Fz-Pz montage was the optimal configuration regardless of the choice of the conductivity values (Supplementary Table S1). Thus, the FCz-POz configuration, visualized in Figure 1, was used in the experiment.

### 2.5 Sit-to-stand training

The participants performed the sit-to-stand task starting from a seated position on a 20 cm box. They were instructed to perform the sit-to-stand task by standing up straight from the box as quickly as possible with fully extended trunk, hip joints, and knee joints, then sit down. The training protocol of a single session consisted of 5 sets of 1 min sit-to-stand task with a 180-s rest period between sets (Figure 2). While monitoring the participant’s performance to ensure proper form (fully extend the trunk, hip joints, and knee joints) examiners counted the completion of each correctly executed sit-to-stand. The counts were averaged over the 5 sets. The sit-to-stand training was conducted a total of 5 sessions. The 48 to 72 h interval between sessions was used to avoid the effect of fatigue on the performance. Before training session, participants warmed up on a bicycle for 5 min, then performed a set of stretches focused on the knee extensor and flexor muscles. They repeated the same routine to warm down after each session.

### 2.6 Muscle strength assessment

Knee extensor and flexor peak torques were assessed using an isokinetic dynamometer (Multi-Joint 3, Biodex Medical Systems Inc., Shirley, NY, United States) with the same procedure as Maeda et al. (2017). Participants were instructed to extend the knee with maximal effort while the dynamometer flexed the knee at a speed of 30°/s from the initial 20° to the 90° eccentric contraction of the knee extensors. For the measurement of the knee flexors torques, participants flexed the knee while the dynamometer extended the knee at 30°/s from 90° to 20°. The maximal knee extensor and flexor torques were evaluated for 3 sets (5 repetitions/set) under eccentric (30°/s) conditions. The maximal knee extensor and flexor torques obtained in 5 repetitions were taken as the peak torque, and the average of the peak torque for each set was calculated.

### 2.7 Electromyography

The participants were comfortably seated in a chair with their arms resting on a cushion. The electromyography (EMG) was recorded via Ag/AgCl-plated surface electrodes (1 cm diameter) placed 2 cm apart over the right rectus femoris (RF) muscle. Responses were acquired using a Neuropack MEB-2200 system (Nihon Kohden, Tokyo, JPN) filtered in the 10 Hz to 1 kHz pass-band. EMG signals were sampled at 5 kHz and stored on the computer for off-line analysis using the LabVIEW software (National Instruments Inc., Austin, Texas, United States).

### 2.8 Transcranial magnetic stimulation

To assess changes in motor cortex excitability, single-pulse transcranial magnetic stimulation (TMS) was applied to the leg area of the left M1 using a magnetic stimulator (Magstim200, Magstim, Dyfed, UK) connected to a double-cone coil of 110-mm diameter. The hotspot of the M1 was confirmed based on induction of the largest MEP amplitude in the right RF muscle during tonic voluntary contraction. The stimulation intensity was adjusted to 120% of the active motor threshold (aMT). The aMT was defined as the lowest stimulus intensity needed to produce MEPs greater than 200 μV in at least 5 out of 10 consecutive trials during the maintenance of 100 μV of RF voluntary isometric contraction (Rossini et al., 2015; Temesi et al., 2017). The time between stimulus pulses was varied between 5 and 7 s. The stimulus timing was automatically controlled using LabVIEW.

In order to induce SICI we used a subthreshold conditioning paired-pulse paradigm (Kujirai et al., 1993). We used stimulus intensities of 80% aMT for the conditioning stimulus and 120% resting motor threshold (rMT) for the test stimulus. Throughout the experiment, the test stimulus was adjusted to maintain the MEP amplitude equal to the RF MEP amplitude at baseline. The interstimulus interval was set at 2.5 ms, and 15 MEPs were recorded from the RF muscle (Fisher et al., 2002). The conditioned MEP amplitudes were expressed as percentages of the mean test MEP amplitudes.

### 2.9 Statistical analysis

The primary outcome measures included the sit-to-stand counts and muscle strength measured pre-training and post-training. We used the 2-way mixed-model analysis of variance (ANOVA) to evaluate the differences in outcome with group (A-P tDCS, P-A tDCS, sham tDCS) and time (pre-training and post-training) used as within-subject factors. Muscle strength was tested separately for the left and right knee flexors and extensors. Similarly, for the sit-to-stand counts during training sessions, we used a 2-way mixed-model ANOVA with the group (A-P tDCS, P-A tDCS, sham tDCS) and time (pre-training, session 1, session 2, session 3, session 4, and session 5) as factors. A t-test with Bonferroni adjustment for multiple comparisons was performed to compare training effects for the group and time factor. For MEP amplitudes and SICI, we applied a 2-way mixed-model ANOVA with the group (A-P tDCS, P-A tDCS, sham tDCS) and time (pre-training, immediately after a first session of training, post-training) as factors. A t-test with Bonferroni adjustment for multiple comparisons was performed to compare changes in cortical excitability pre-training, immediately after the first session of training, post-training for the group and time factor. *p* values <0.05 were considered statistically significant for all analyses. Statistical analyses were performed using SPSS 28.0 (IBM, Armonk, NY, United States).

## 3 Results

All participants successfully completed the 3-week training. There were no reports of adverse events related to the training or tDCS. However, data for one participant in the P-A tDCS group was lost due to a device malfunction during the post-training test MEP and SICI measurements. Despite this, the percentage of correct responses in the condition remained below the chance level, indicating that, blinding in the intervention condition was maintained. The results of the motor performance and physiological factors for each group are illustrated in Tables 3, 4.

**Table 3:** Motor performance.

	A-P tDCS group (*n* = 12)		P-A tDCS group (*n* = 12)		sham tDCS group (*n* = 12)	
	Pre	Post	Pre	Post	Pre	Post
SIT-to-stand counts						
	43 (9)	60 (4)	44 (11)	55 (6)	45 (7)	54 (6)
KNEE extensor torque (Nm)						
Right side	99.9 (19.8)	95.9 (25.0)	102.4 (31.9)	107.9 (28.1)	97.2 (25.9)	98.7 (28.5)
Left side	87.0 (26.0)	87.4 (24.5)	95.0 (32.6)	100.3 (30.6)	89.8 (24.5)	86.3 (21.7)
KNEE flexor torque (Nm)						
Right side	128.3 (22.2)	124.8 (20.4)	124.2 (20.3)	144.2 (19.9)	117.6 (26.2)	123.1 (28.9)
Left side	123.0 (24.3)	121.8 (24.7)	122.5 (18.7)	145.3 (20.5)	117.0 (16.0)	116.3 (24.8)

Data are presented as the mean (standard deviation). The data demonstrate the sit-to-stand counts and muscle strength before training (Pre) and after 3 weeks training (Post). tDCS, transcranial direct current stimulation; A-P tDCS, anterior-to-posterior current flow tDCS; P-A tDCS, posterior-to-anterior current flow tDCS.

**Table 4:** Physiological factors.

A-P tDCS group (*n* = 12)			P-A tDCS group (*n* = 12)			sham tDCS group (*n* = 12)		
Pre	S1	Post	Pre	S1	Post	Pre	S1	Post
SICI (% of test MEP)								
46.2 (14.5)	41.3 (16.7)	47.5 (18.7)	45.5 (11.2)	69.9 (10.4)	62.3 (18.4)	46.6 (19.4)	52.9 (14.7)	50.5 (26.1)
MEP AMPLITUDES (mV)								
1.44 (0.86)	0.96 (0.83)	1.15 (0.79)	1.49 (0.76)	1.30 (0.83)	1.70 (1.51)	1.42 (0.72)	1.53 (0.87)	1.63 (0.66)

Data are presented as the mean (standard deviation). The data show the short-interval intracortical inhibition (SICI) and motor-evoked potential (MEP) before training (Pre), after session 1 (S1), and after 3 weeks training (Post). tDCS, transcranial direct current stimulation; A-P tDCS, anterior-to-posterior current flow tDCS; P-A tDCS, posterior-to-anterior current flow tDCS.

### 3.1 The sit-to-stand counts

**Figure 3:**
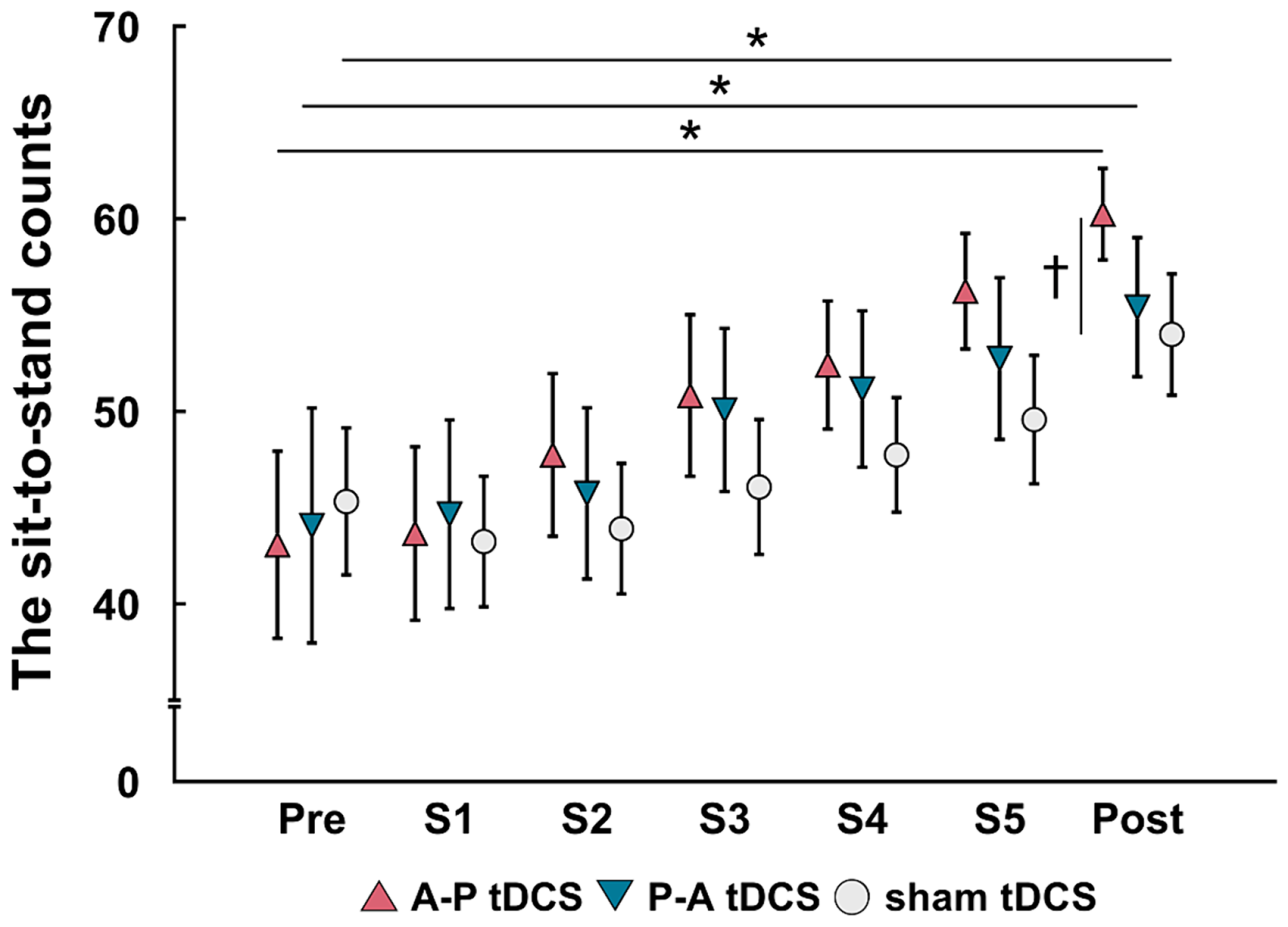
Changes in the sit-to-stand counts following tDCS combined with sit-to-stand training. Data are presented as mean (symbols) ± the confidence interval (whiskers). Data for the three groups are shown in different symbols: A-P tDCS (red triangles); P-A tDCS (blue inverted triangles); and sham tDCS (gray circles). The sit-to-stand counts were assessed before training (Pre), session 1 (S1), session 2 (S2), session 3 (S3), session 4 (S4), session 5 (S5) and after 3 weeks training (Post). Significant (*p* < 0.05) pairwise differences within group between the pre and another time point (asterisk) and between groups at a fixed time point (dagger) are indicated.

The sit-to-stand counts continued to increase throughout the 5 sessions and were greater after than before training in all groups. At post-training assessment, the sit-to-stand counts for the A-P tDCS group were significantly higher than the sham tDCS group, indicating that A-P tDCS promoted sit-to-stand performance (Figure 3). These observations were supported by primary outcome results of the 2-way mixed-model ANOVA, which revealed significant interactions (*F*_(2, 33)_ = 3.652, *p* < 0.05), with a significant main effect of time (*F*_(1, 33)_ = 88.990, *p* < 0.01). No significant main effect of the intervention was observed (F_(2, 33)_ = 0.398, *p* = 0.675). Similarly, the multiple comparison test revealed that the sit-to-stand counts were significantly increased at post-training for all groups compared to the pre-training (*p* < 0.01). When the sit-to-stand counts were compared between the groups, the counts were significantly greater in the A-P tDCS group compared to the sham tDCS group (*p* < 0.05) at post-training. However, 2-way mixed-model ANOVA found no significant differences in the counts among the groups during training session (*F*_(10, 165)_ = 1.879, *p* = 0.051). The main effects of time were significant (*F*_(5, 165)_ = 25.685, *p* < 0.01), but no significant main effect of the intervention was observed (F_(2, 33)_ = 0.777, *p* = 0.468).

### 3.2 Muscle strength

**Figure 4:**
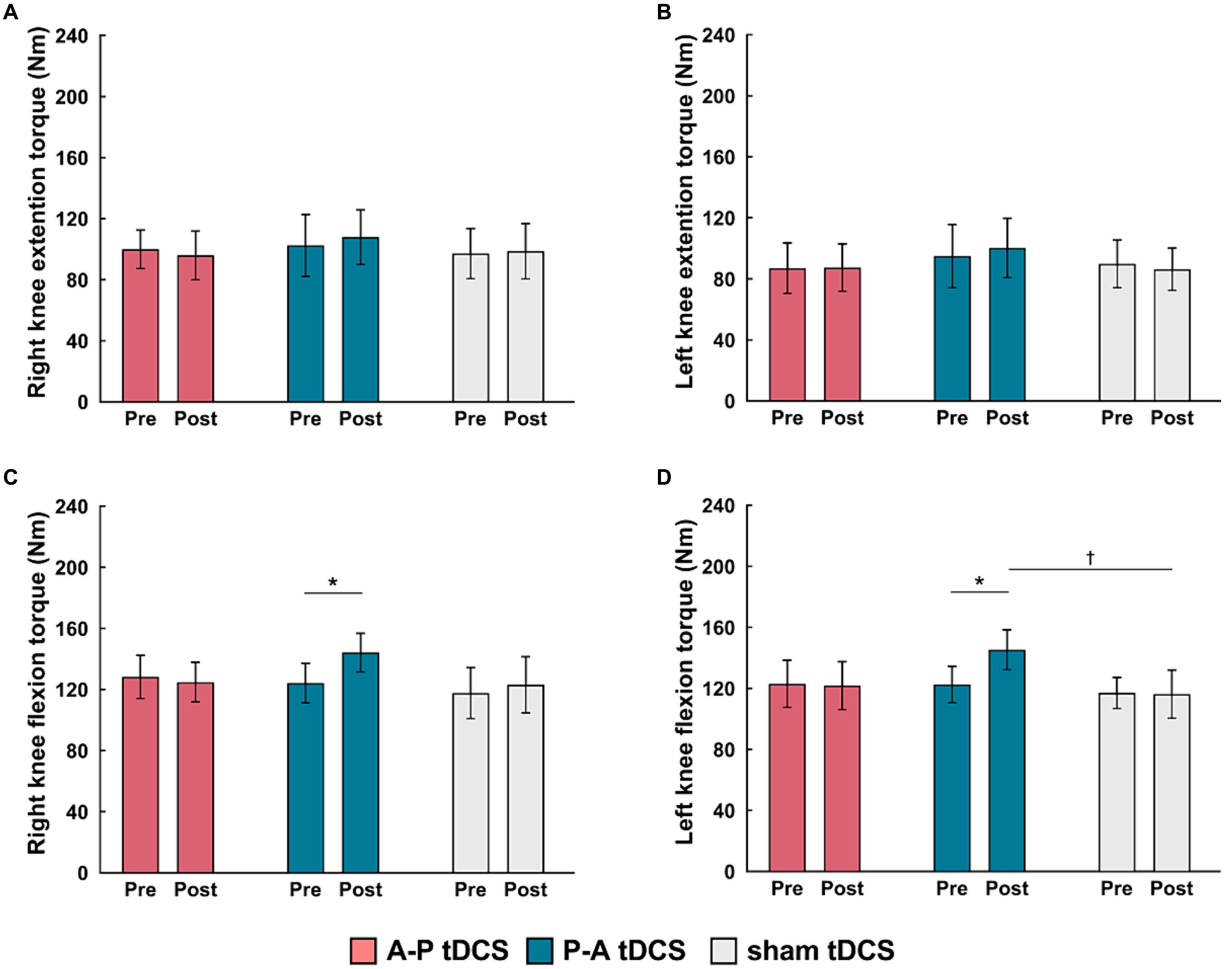
Changes in muscle strength following tDCS combined with sit-to-stand training. Muscle torque (Nm) measurements are presented as mean (bar) ± the confidence interval (whiskers) for the **(A)** right knee extension, **(B)** left knee extension, **(C)** right knee flexion, and **(D)** left knee flexion. Data for the three groups are labeled by color: A-P tDCS (red), P-A tDCS (blue), sham tDCS (gray). Muscle torque were assessed before training (Pre) and after 3 weeks training (Post). Significant (*p* < 0.05) pairwise differences within group between the Pre and Post (asterisks) and between groups at a fixed time point (daggers) are indicated.

The peak torque in right and left knee flexors were increased in the P-A tDCS group, and only in that group, following 3 weeks of training (Figure 4). In contrast, no changes were observed for peak torque in the knee extensors in any of the groups. Statistical analyses supported these observations. For knee flexor torques, 2-way mixed-model ANOVA showed significant interactions between intervention and time (Right: *F*_(2, 33)_ = 8.099, *p* < 0.01, Left: F_(2, 33)_ = 10.917, *p* < 0.01). The main effects of time were significant (Right: F_(1, 33)_ = 9.425, *p* < 0.01, Left: F_(1, 33)_ = 8.436, *p* < 0.01). No significant main effects of the intervention were observed (*p* > 0.1 for both right and left). For knee extensor torques, there was also no interaction between intervention and time, and the main effects of both time and intervention were not significant (*p* > 0.1 for each test, for both right and left).

Multiple comparison test revealed that the left and right knee flexor peak torques in the P-A tDCS group were significantly increased at post-training above their pre-training levels (*p* < 0.01). The left knee flexor peak torques in the P-A tDCS group, in particular, were significantly increased over levels in the sham tDCS group at post-training (*p* < 0.05).

### 3.3 MEP amplitudes

There was no significant interaction between intervention and time (*F*_(4, 66)_ = 0.655, *p* = 0.626), and the main effects of both time and intervention were not significant (*p* > 0.3 for both). These results confirmed that MEP amplitudes remained stable at pre-training levels during and after training.

#### 3.3.1 SICI

**Figure 5:**
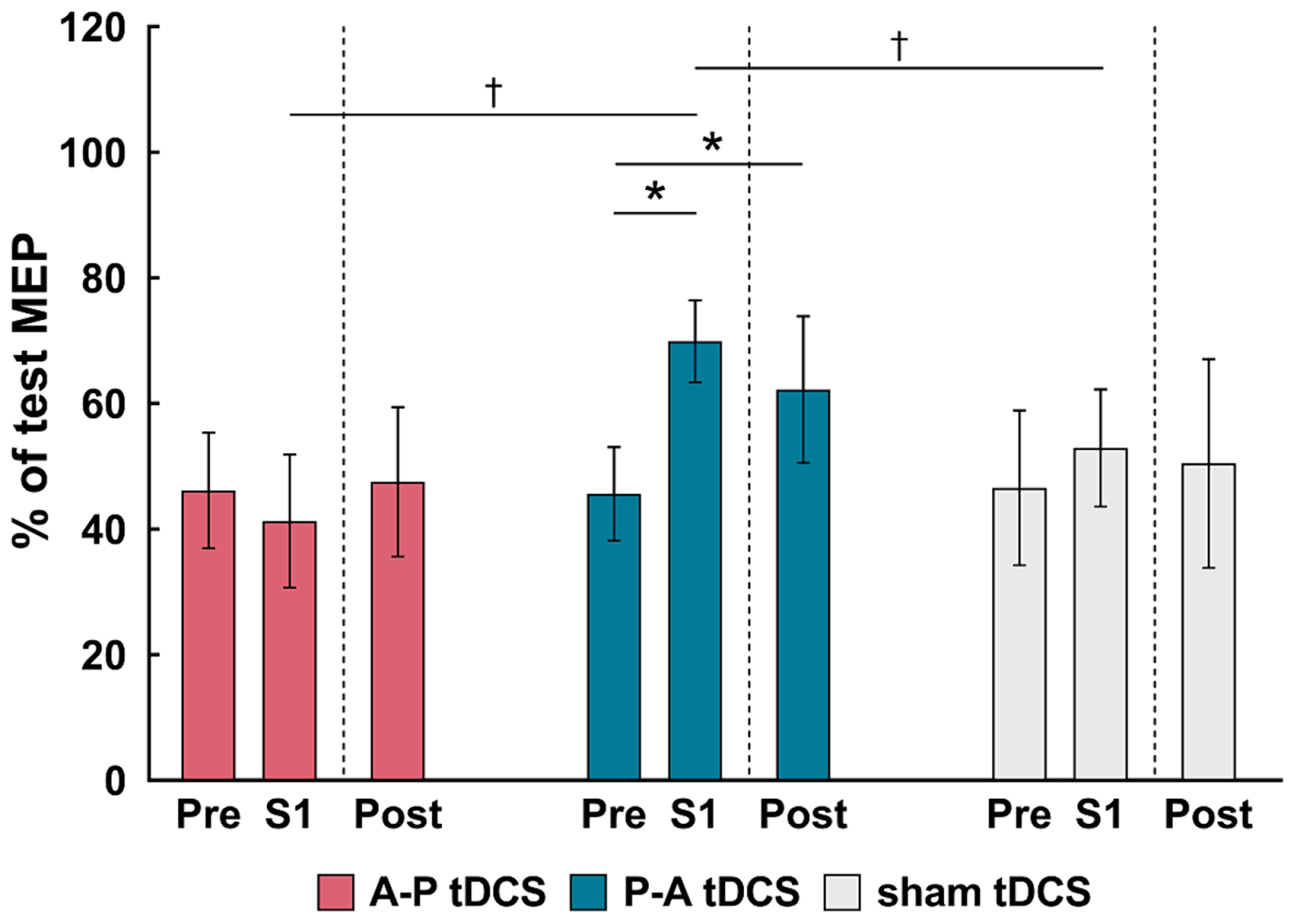
Changes in the short-interval intracortical inhibition (SICI) following tDCS combined with sit-to-stand training. The relative changes in SICI, as a % of the reference (test MEP), are shown as the mean (bars) and confidence intervals (whiskers) for three time points (pre, S1 and Post) in different colors for the three groups: A-P tDCS (red), P-A tDCS (blue), sham tDCS (gray). SICI were assessed before training (Pre), after session 1 (S1) and after 3 weeks training (Post). Significant (*p* < 0.05) pairwise differences within group between the pre and another time point (asterisks) and between groups at a fixed time point (dagger) are indicated.

SICI was suppressed in the P-A tDCS group immediately after the first session of training and remained so post-training. Moreover, compared to other groups, the suppression seen in the P-A tDCS group was marked (Figure 5), suggesting that P-A tDCS-induced plastic changes of SICI in the primary motor cortex.

This result were borne out by a 2-way mixed-model ANOVA, showing significant interactions (*F*_(4, 65)_ = 3.261, *p* < 0.05) between intervention and time, and main effects of session times (*F*_(2, 65)_ = 3.796, *p* < 0.05) and intervention (F_(2, 33)_ = 3.678, *p* < 0.05). Multiple comparison showed that SICI in P-A tDCS group was significantly decreased immediately after the first session of training and remained so post-training below the pre-training values (both, *p* < 0.05). Furthermore, the SICI was significantly decreased in the P-A tDCS group compared to the A-P and sham tDCS groups immediately after the first session of training (both, *p* < 0.05).

## 4 Discussion

This is the first study to investigate the effects of different current flows with electrode placement optimized for tDCS to produce maximum EFs in SMA and M1. Our primary findings are as follows: (1) A-P tDCS enhanced sit-to-stand counts after 3 weeks of training; (2) P-A tDCS increased the right and left knee flexor peak torques after 3 weeks of training; and (3) P-A tDCS decreased SICI immediately after the first session of training held it decreased until post-training. These results indicate that optimized electrode placement of tDCS can promote motor performances and modulate cortical excitability depending on the current flow. Moreover, all participants completed the training without adverse effects, making applying our method in healthy controls a valuable step toward enhancing current neurorehabilitation practices.

The conventional method of placing tDCS electrodes is just above the target brain area where researchers expected to modulate cortical excitability (Nitsche and Paulus, 2000; Tanaka et al., 2009, 2011). However, this strategy for electrodes placement was unable to induce optimal EFs in the target cortical areas (Laakso et al., 2016), and it often fails to improve motor function and modulate cortical excitability in healthy individuals and patients with stroke (Kan et al., 2013; Muthalib et al., 2013; Maeda et al., 2017; Klomjai et al., 2018; Alix-Fages et al., 2020; Katagiri et al., 2021). Therefore, we adopted FCz and POz for the placement of the two electrodes because these loci were identified, by simulated EFs in MRI-based model brains, as those leading to the inducing of maximum EFs to the SMA and M1. The conventional method of placing the stimulation electrode just above the target brain area (i.e., Cz plus a reference electrode at a distant location) was not optimal for inducing EF to the target brain area (Table 1). The results of these simulations indicated that unconventional electrode placement (i.e., where the target brain area is between the two electrodes) may modulate the brain area. However, to the best of our knowledge, no studies have examined changes in motor performance with optimized electrode placement tDCS by electric field simulation. A previous study reported a positive correlation between the EF values induced in the target brain areas and changes in cortical excitability caused by tDCS (Mosayebi-Samani et al., 2021). Therefore, use of electrical field simulations for electrode placement tDCS may modulate cortical excitability and enhance motor performance. In addition, studies have reported that the conventional tDCS targeting M1 combined with long-term muscle strength training did not improve muscle strength or motor performance (Hendy and Kidgell, 2013; Maeda et al., 2017; Marcos-Frutos et al., 2023; Jung et al., 2024). On the other hand, we found that optimized electrode placement by electric field simulation enhances muscle strength and motor performance. Therefore, optimized electrode placement tDCS might elicit improvements in muscle strength and motor performance that could not be achieved with conventional methods. These results suggest that the determination of tDCS electrode placement by electric field simulation for standard brain models may provide important findings for future neurorehabilitation studies.

Surprisingly, we found that motor cortex excitability and motor performance improved when currents flowed in opposite directions. The tDCS effect depends on the relationship between the EF vector and the morphology and orientation of the neurons and individual neuronal compartments, which determines the polarization state of neurons (Liu et al., 2018). Indeed, it has been reported that corticospinal excitability and motor task learning are affected by current flow between the tDCS electrodes (Rawji et al., 2018; Hannah et al., 2019). Our tDCS electrode placement produced a similarly strong EFs in SMA and M1. Therefore, different populations of neurons in SMA and M1 may have depolarized or hyperpolarized depending on the direction of the current flow. A-P tDCS in our experiments induced current flows from SMA to M1, which is expected to effectively depolarize in the SMA area. This in turn is thought to facilitate the coordination and execution of motor programs during skilled movement and postural control, which are functions of the SMA (Mihara et al., 2008, 2012; Fujimoto et al., 2014). Investigators using a similar electrode placement to ours have reported that body weight-supported treadmill training and tDCS with anode in front of Cz and cathode over inion improved the balance and gait function after stroke, but paradoxically without changes in leg motor function (Manji et al., 2018). Conversely, P-A tDCS induces current from M1 to SMA, which is expected to effectively depolarize in the M1 area. In the previous studies, posterior to anterior current flow from M1 to the opposite supraorbital improved muscle strength in the upper and lower limbs (Tanaka et al., 2009; Hazime et al., 2017; Vargas et al., 2018). Therefore, posterior to anterior current flow targeting M1 and SMA in the P-A tDCS group may have led to the increased peak torque of knee flexion after the 3 weeks of training.

Interestingly, we found that the peak torque was improved in knee flexors, but not in the knee extensor. During the sit-to-standing exercise, knee flexor muscles are required for the smooth extension of the knee joint via eccentric muscle contraction, whereas knee extensor muscles are activated to extend the knee joint via concentric muscle contraction (Bryanton and Bilodeau, 2017). The effectiveness of muscle training depends on the mode of muscle contraction being evaluated (Higbie et al., 1996). Based on these earlier studies, the muscle strength increases we detected in the assessment of eccentric contraction of the knee flexor muscles was not unexpected.

A decrease in SICI was observed in the P-A tDCS group alone, while SICI did not change in the other groups. This result may also support the hypothesis that different populations of neurons in SMA and M1 may have depolarized or hyperpolarized depending on current flow. It has been reported that conventional non-optimized anodal tDCS over M1 reduced SICI there (Nitsche et al., 2005; Biabani et al., 2018). Others have shown that changes in SICI probably reflect the activity of GABA_A_-ergic intracortical inhibitory connections in cortical layer 1 since the inhibition is evoked by conditioning stimulus below motor threshold (Di Lazzaro et al., 2006; Di Lazzaro and Rothwell, 2014). In addition, previous studies suggested that the current of tDCS will preferentially polarize neural components that are aligned with the direction of current flow (Bikson et al., 2004; Jackson et al., 2016; Hannah et al., 2019). Therefore, the neural elements involved in the change in SICI were more likely to be selectively modulated according to the direction of the applied EFs because they were specific to our task and located in a shallow layer. This is speculation that change in SICI might be associated with increasing the efficiency of transmission of the descending drive of M1, resulting in stronger muscle contraction (Hendy and Kidgell, 2014; Hendy et al., 2015). In contrast, the effects of the different tDCS conditions on corticospinal excitability were not observed in the present study. An earlier review reported for conventional anodal tDCS an increase in corticospinal excitability along with the reduction in SICI (McNeil et al., 2009). One possible factor is fatigue after training. It is known that fatigue after muscle contractions inhibits the corticospinal response at the spinal level (McNeil et al., 2009, 2011; Carroll et al., 2017). Thus, a negative effect on corticospinal rather than intracortical excitability may have been at play.

Relearning the sit-to-stand movement is essential to rehabilitation after a stroke (Alexander et al., 2000). Conventional rehabilitation, combined with anodal tDCS over M1, improved sit-to-stand performance in patients following stroke (Andrade et al., 2017). Therefore, tDCS with optimized electrode placement may assist with stroke rehabilitation. Furthermore, our results indicate that different effects are observed relative to tDCS current flow direction. These findings provide new insights into current neurorehabilitation paradigms. Specifically, the A-P tDCS could be adapted for stroke patients needing to improve their sit-to-stand performance. Additionally, P-A tDCS could be adapted for stroke patients requiring enhanced muscle strengthening. Therefore, the optimized electrode placement for tDCS can be determined based on patient-specific treatment objectives.

However, this study has several limitations. First, we have not compared the effects of the optimized electrode placement with those of the conventional tDCS electrode placement. A single session of conventional tDCS combined with exercise has been shown to enhance muscle strength and modulate corticospinal tract excitability (Kim and Ko, 2013; Washabaugh et al., 2016). In contrast, the effects of repetitive sessions remain unclear (Wang et al., 2021; Marcos-Frutos et al., 2023). Therefore, further studies are necessary to compare the effects of optimized electrode placement tDCS to conventional tDCS in repetitive sessions. Second, the cortical EFs were calculated numerically through the FEM in an anatomical model in accordance with the 62 MRIs, not the participants that were recruited. Third, the SMA activity changes were not evaluated following the training. Fourth, this study was conducted on healthy individuals. Therefore, in the future, EFs need to be calculated for each subject to reduce inter-individual variability in tDCS-induced effects. Additionally, SMA activity changes after training should be evaluated through postural control tasks. Finally, further research is required to examine whether tDCS with an optimized electrode placement can improve motor performance and brain function more than the conventional tDCS method in patients with stroke. In conclusion, we showed that electrode placement of the maximal EFs in SAM and M1 estimated by electric field simulation enhances sit-to-stand performance, lower limb muscle strength, and modulates motor cortical excitability depending on the direction of current flow in young healthy individuals.

## Data availability statement

The original contributions presented in the study are included in the article/Supplementary material, further inquiries can be directed to the corresponding author.

## Ethics statement

The studies involving humans were approved by Yamagata Prefectural University of Health Sciences (approval number: 1806-06). The studies were conducted in accordance with the local legislation and institutional requirements. The participants provided their written informed consent to participate in this study.

## Author contributions

TS: Conceptualization, Data curation, Investigation, Writing – original draft. NK: Data curation, Investigation, Writing – original draft. SS: Conceptualization, Data curation, Investigation, Writing – original draft. IL: Formal analysis, Writing – review & editing. ShT: Software, Writing – review & editing. RO: Formal analysis, Writing – review & editing. SaT: Conceptualization, Funding acquisition, Writing – review & editing. TY: Conceptualization, Data curation, Formal analysis, Funding acquisition, Methodology, Supervision, Writing – original draft, Writing – review & editing.

## References

[ref1] Alexander N. B. Galecki A. T. Nyquist L. V. Hofmeyer M. R. Grunawalt J. C. Grenier M. L.. (2000). Chair and bed rise performance in ADL-impaired congregate housing residents. J. Am. Geriatr. Soc. 48, 526–533. doi: 10.1111/j.1532-5415.2000.tb04999.x, PMID: 10811546

[ref2] Alix-Fages C. García-Ramos A. Calderón-Nadal G. Colomer-Poveda D. Romero-Arenas S. Fernández-Del-Olmo M.. (2020). Anodal transcranial direct current stimulation enhances strength training volume but not the force-velocity profile. Eur. J. Appl. Physiol. 120, 1881–1891. doi: 10.1007/s00421-020-04417-2 32533243

[ref3] Andrade S. M. Ferreira J. J. A. Rufino T. S. Medeiros G. Brito J. D. da Silva M. A.. (2017). Effects of different montages of transcranial direct current stimulation on the risk of falls and lower limb function after stroke. Neurol. Res. 39, 1037–1043. doi: 10.1080/01616412.2017.1371473 28885111

[ref4] Angius L. Mauger A. R. Hopker J. Pascual-Leone A. Santarnecchi E. Marcora S. M. (2018). Bilateral extracephalic transcranial direct current stimulation improves endurance performance in healthy individuals. Brain Stimul. 11, 108–117. doi: 10.1016/j.brs.2017.09.017, PMID: 29079458 PMC6298602

[ref5] Angius L. Pageaux B. Hopker J. Marcora S. M. Mauger A. R. (2016). Transcranial direct current stimulation improves isometric time to exhaustion of the knee extensors. Neuroscience 339, 363–375. doi: 10.1016/j.neuroscience.2016.10.028, PMID: 27751960

[ref6] Biabani M. Aminitehrani M. Zoghi M. Farrell M. Egan G. Jaberzadeh S. (2018). The effects of transcranial direct current stimulation on short-interval intracortical inhibition and intracortical facilitation: a systematic review and meta-analysis. Rev. Neurosci. 29, 99–114. doi: 10.1515/revneuro-2017-0023, PMID: 28820738

[ref7] Bikson M. Inoue M. Akiyama H. Deans J. K. Fox J. E. Miyakawa H.. (2004). Effects of uniform extracellular DC electric fields on excitability in rat hippocampal slices in vitro. J. Physiol. 557, 175–190. doi: 10.1113/jphysiol.2003.055772, PMID: 14978199 PMC1665051

[ref8] Bryanton M. Bilodeau M. (2017). The role of thigh muscular efforts in limiting sit-to-stand capacity in healthy young and older adults. Aging Clin. Exp. Res. 29, 1211–1219. doi: 10.1007/s40520-016-0702-7, PMID: 28238153

[ref9] Carroll T. J. Taylor J. L. Gandevia S. C. (2017). Recovery of central and peripheral neuromuscular fatigue after exercise. J. Appl. Physiol. 122, 1068–1076. doi: 10.1152/japplphysiol.00775.2016 27932676

[ref10] Chang M. C. Kim D. Y. Park D. H. (2015). Enhancement of cortical excitability and lower limb motor function in patients with stroke by transcranial direct current stimulation. Brain Stimul. 8, 561–566. doi: 10.1016/j.brs.2015.01.411 25736569

[ref11] Chew T. Ho K. A. Loo C. K. (2015). Inter- and intra-individual variability in response to transcranial direct current stimulation (tDCS) at varying current intensities. Brain Stimul. 8, 1130–1137. doi: 10.1016/j.brs.2015.07.031, PMID: 26294061

[ref12] Dale A. M. Fischl B. Sereno M. I. (1999). Cortical surface-based analysis I segmentation and surface reconstruction. NeuroImage 9, 179–194. doi: 10.1006/nimg.1998.0395 9931268

[ref13] Di Lazzaro V. Pilato F. Dileone M. Ranieri F. Ricci V. Profice P.. (2006). GABAA receptor subtype specific enhancement of inhibition in human motor cortex. J. Physiol. 575, 721–726. doi: 10.1113/jphysiol.2006.114694, PMID: 16809358 PMC1995685

[ref14] Di Lazzaro V. Rothwell J. C. (2014). Corticospinal activity evoked and modulated by non-invasive stimulation of the intact human motor cortex. J. Physiol. 592, 4115–4128. doi: 10.1113/jphysiol.2014.274316, PMID: 25172954 PMC4215763

[ref15] Evans C. Bachmann C. Lee J. S. A. Gregoriou E. Ward N. Bestmann S. (2020). Dose-controlled tDCS reduces electric field intensity variability at a cortical target site. Brain Stimul. 13, 125–136. doi: 10.1016/j.brs.2019.10.004 31653475

[ref16] Fischl B. Dale A. M. (2000). Measuring the thickness of the human cerebral cortex from magnetic resonance images. Proc. Natl. Acad. Sci. USA 97, 11050–11055. doi: 10.1073/pnas.200033797, PMID: 10984517 PMC27146

[ref17] Fischl B. Sereno M. I. Tootell R. B. Dale A. M. (1999). High-resolution intersubject averaging and a coordinate system for the cortical surface. Hum. Brain Mapp. 8, 272–284. doi: 10.1002/(sici)1097-0193(1999)8:4<272::aid-hbm10>3.0.co;2-4, PMID: 10619420 PMC6873338

[ref18] Fisher R. J. Nakamura Y. Bestmann S. Rothwell J. C. Bostock H. (2002). Two phases of intracortical inhibition revealed by transcranial magnetic threshold tracking. Exp. Brain Res. 143, 240–248. doi: 10.1007/s00221-001-0988-2, PMID: 11880900

[ref19] Fonov V. Evans A. C. Botteron K. Almli C. R. McKinstry R. C. Collins D. L.. (2011). Unbiased average age-appropriate atlases for pediatric studies. NeuroImage 54, 313–327. doi: 10.1016/j.neuroimage.2010.07.033, PMID: 20656036 PMC2962759

[ref20] Fonov V. Evans A. C. McKinstry R. C. Almli C. R. Collins D. L. (2009). Unbiased nonlinear average age-appropriate brain templates from birth to adulthood. NeuroImage 47:S102. doi: 10.1016/S1053-8119(09)70884-5

[ref21] Fujimoto H. Mihara M. Hattori N. Hatakenaka M. Kawano T. Yagura H.. (2014). Cortical changes underlying balance recovery in patients with hemiplegic stroke. NeuroImage 85, 547–554. doi: 10.1016/j.neuroimage.2013.05.014, PMID: 23684871

[ref22] Fujimoto S. Tanaka S. Laakso I. Yamaguchi T. Kon N. Nakayama T.. (2017). The effect of dual-hemisphere transcranial direct current stimulation over the parietal operculum on tactile orientation discrimination. Front. Behav. Neurosci. 11:173. doi: 10.3389/fnbeh.2017.00173, PMID: 28979197 PMC5611440

[ref23] Guerra A. López-Alonso V. Cheeran B. Suppa A. (2020). Variability in non-invasive brain stimulation studies: reasons and results. Neurosci. Lett. 719:133330. doi: 10.1016/j.neulet.2017.12.058, PMID: 29294333

[ref24] Hannah R. Iacovou A. Rothwell J. C. (2019). Direction of TDCS current flow in human sensorimotor cortex influences behavioural learning. Brain Stimul. 12, 684–692. doi: 10.1016/j.brs.2019.01.016, PMID: 30738775 PMC6491497

[ref25] Hazime F. A. da Cunha R. A. Soliaman R. R. Romancini A. C. B. Pochini A. C. Ejnisman B.. (2017). Anodal transcranial direct current stimulation (TDCS) increases isometric strength of shoulder rotators muscles in handball PLAYERS. Int. J. Sports Phys. Ther. 12, 402–407. Available at: https://ijspt.org/. 28593094 PMC5455189

[ref26] Hendy A. M. Kidgell D. J. (2013). Anodal tDCS applied during strength training enhances motor cortical plasticity. Med. Sci. Sports Exerc. 45, 1721–1729. doi: 10.1249/MSS.0b013e31828d2923, PMID: 23470308

[ref27] Hendy A. M. Kidgell D. J. (2014). Anodal-tDCS applied during unilateral strength training increases strength and corticospinal excitability in the untrained homologous muscle. Exp. Brain Res. 232, 3243–3252. doi: 10.1007/s00221-014-4016-8, PMID: 24942703

[ref28] Hendy A. M. Teo W. P. Kidgell D. J. (2015). Anodal transcranial direct current stimulation prolongs the cross-education of strength and Corticomotor plasticity. Med. Sci. Sports Exerc. 47, 1788–1797. doi: 10.1249/MSS.0000000000000600, PMID: 25551405

[ref29] Higbie E. J. Cureton K. J. Warren G. L. Prior B. M. (1996). Effects of concentric and eccentric training on muscle strength, cross-sectional area, and neural activation. J. Appl. Physiol. 81, 2173–2181. doi: 10.1152/jappl.1996.81.5.2173, PMID: 8941543

[ref30] Jackson M. P. Rahman A. Lafon B. Kronberg G. Ling D. Parra L. C.. (2016). Animal models of transcranial direct current stimulation: methods and mechanisms. Clin. Neurophysiol. 127, 3425–3454. doi: 10.1016/j.clinph.2016.08.016, PMID: 27693941 PMC5083183

[ref31] Jeffery D. T. Norton J. A. Roy F. D. Gorassini M. A. (2007). Effects of transcranial direct current stimulation on the excitability of the leg motor cortex. Exp. Brain Res. 182, 281–287. doi: 10.1007/s00221-007-1093-y 17717651

[ref32] Julious S. A. (2005). Sample size of 12 per group rule of thumb for a pilot study. Pharm. Stat. 4, 287–291. doi: 10.1002/pst.185

[ref33] Jung J. Salazar Fajardo J. C. Kim S. Kim B. Oh S. Yoon B. (2024). Effect of tDCS combined with physical training on physical performance in a healthy population. Res. Q. Exerc. Sport 95, 149–156. doi: 10.1080/02701367.2023.2166894, PMID: 37036388

[ref34] Kan B. Dundas J. E. Nosaka K. (2013). Effect of transcranial direct current stimulation on elbow flexor maximal voluntary isometric strength and endurance. Appl. Physiol. Nutr. Metab. 38, 734–739. doi: 10.1139/apnm-2012-0412, PMID: 23980731

[ref35] Katagiri N. Kawakami S. Okuyama S. Koseki T. Kudo D. Namba S.. (2021). Single-session cerebellar transcranial direct current stimulation affects postural control learning and cerebellar brain inhibition in healthy individuals. Cerebellum 20, 203–211. doi: 10.1007/s12311-020-01208-5, PMID: 33108574

[ref36] Kim G. W. Ko M. H. (2013). Facilitation of corticospinal tract excitability by transcranial direct current stimulation combined with voluntary grip exercise. Neurosci. Lett. 548, 181–184. doi: 10.1016/j.neulet.2013.05.037 23726882

[ref37] Klomjai W. Aneksan B. Pheungphrarattanatrai A. Chantanachai T. Choowong N. Bunleukhet S.. (2018). Effect of single-session dual-tDCS before physical therapy on lower-limb performance in sub-acute stroke patients: a randomized sham-controlled crossover study. Ann. Phys. Rehabil. Med. 61, 286–291. doi: 10.1016/j.rehab.2018.04.005, PMID: 29763676

[ref38] Kujirai T. Caramia M. D. Rothwell J. C. Day B. L. Thompson P. D. Ferbert A.. (1993). Corticocortical inhibition in human motor cortex. J. Physiol. 471, 501–519. doi: 10.1113/jphysiol.1993.sp019912, PMID: 8120818 PMC1143973

[ref39] Laakso I. Hirata A. (2012). Fast multigrid-based computation of the induced electric field for transcranial magnetic stimulation. Phys. Med. Biol. 57, 7753–7765. doi: 10.1088/0031-9155/57/23/7753, PMID: 23128377

[ref40] Laakso I. Mikkonen M. Koyama S. Hirata A. Tanaka S. (2019). Can electric fields explain inter-individual variability in transcranial direct current stimulation of the motor cortex? Sci. Rep. 9:626. doi: 10.1038/s41598-018-37226-x, PMID: 30679770 PMC6345748

[ref41] Laakso I. Tanaka S. Koyama S. De Santis V. Hirata A. (2015). Inter-subject variability in electric fields of motor cortical tDCS. Brain Stimul. 8, 906–913. doi: 10.1016/j.brs.2015.05.002, PMID: 26026283

[ref42] Laakso I. Tanaka S. Mikkonen M. Koyama S. Sadato N. Hirata A. (2016). Electric fields of motor and frontal tDCS in a standard brain space: a computer simulation study. NeuroImage 137, 140–151. doi: 10.1016/j.neuroimage.2016.05.032, PMID: 27188218

[ref43] Liu A. Vöröslakos M. Kronberg G. Henin S. Krause M. R. Huang Y.. (2018). Immediate neurophysiological effects of transcranial electrical stimulation. Nat. Commun. 9:5092. doi: 10.1038/s41467-018-07233-7, PMID: 30504921 PMC6269428

[ref44] López-Alonso V. Cheeran B. Río-Rodríguez D. Fernández-Del-Olmo M. (2014). Inter-individual variability in response to non-invasive brain stimulation paradigms. Brain Stimul. 7, 372–380. doi: 10.1016/j.brs.2014.02.004, PMID: 24630849

[ref45] López-Alonso V. Fernández-Del-Olmo M. Costantini A. Gonzalez-Henriquez J. J. Cheeran B. (2015). Intra-individual variability in the response to anodal transcranial direct current stimulation. Clin. Neurophysiol. 126, 2342–2347. doi: 10.1016/j.clinph.2015.03.022, PMID: 25922127

[ref46] Madhavan S. Weber K. A. Stinear J. W. (2011). Non-invasive brain stimulation enhances fine motor control of the hemiparetic ankle: implications for rehabilitation. Exp. Brain Res. 209, 9–17. doi: 10.1007/s00221-010-2511-0, PMID: 21170708

[ref47] Maeda K. Yamaguchi T. Tatemoto T. Kondo K. Otaka Y. Tanaka S. (2017). Transcranial direct current stimulation does not affect lower extremity muscle strength training in healthy individuals: a triple-blind Sham-Controlled Study. Front. Neurosci. 11:179. doi: 10.3389/fnins.2017.00179, PMID: 28420959 PMC5378798

[ref48] Manji A. Amimoto K. Matsuda T. Wada Y. Inaba A. Ko S. (2018). Effects of transcranial direct current stimulation over the supplementary motor area body weight-supported treadmill gait training in hemiparetic patients after stroke. Neurosci. Lett. 662, 302–305. doi: 10.1016/j.neulet.2017.10.049, PMID: 29107706

[ref49] Marcos-Frutos D. López-Alonso V. Mera-González I. Sánchez-Molina J. A. Colomer-Poveda D. Márquez G. (2023). Chronic functional adaptations induced by the application of transcranial direct current stimulation combined with exercise programs: a systematic review of randomized controlled trials. J. Clin. Med. 12:6724. doi: 10.3390/jcm12216724, PMID: 37959190 PMC10649950

[ref50] McNeil C. J. Giesebrecht S. Gandevia S. C. Taylor J. L. (2011). Behaviour of the motoneurone pool in a fatiguing submaximal contraction. J. Physiol. 589, 3533–3544. doi: 10.1113/jphysiol.2011.207191, PMID: 21606110 PMC3167116

[ref51] McNeil C. J. Martin P. G. Gandevia S. C. Taylor J. L. (2009). The response to paired motor cortical stimuli is abolished at a spinal level during human muscle fatigue. J. Physiol. 587, 5601–5612. doi: 10.1113/jphysiol.2009.180968, PMID: 19805743 PMC2805373

[ref52] Mihara M. Miyai I. Hatakenaka M. Kubota K. Sakoda S. (2008). Role of the prefrontal cortex in human balance control. NeuroImage 43, 329–336. doi: 10.1016/j.neuroimage.2008.07.029 18718542

[ref53] Mihara M. Miyai I. Hattori N. Hatakenaka M. Yagura H. Kawano T.. (2012). Cortical control of postural balance in patients with hemiplegic stroke. Neuroreport 23, 314–319. doi: 10.1097/WNR.0b013e328351757b, PMID: 22357394

[ref54] Mikkonen M. Laakso I. Tanaka S. Hirata A. (2020). Cost of focality in TDCS: Interindividual variability in electric fields. Brain Stimul. 13, 117–124. doi: 10.1016/j.brs.2019.09.017, PMID: 31606449

[ref55] Montenegro R. A. Midgley A. Massaferri R. Bernardes W. Okano A. H. Farinatti P. (2016). Bihemispheric motor cortex transcranial direct current stimulation improves force steadiness in post-stroke Hemiparetic patients: a randomized crossover controlled trial. Front. Hum. Neurosci. 10:426. doi: 10.3389/fnhum.2016.00426, PMID: 27601988 PMC4994243

[ref56] Mosayebi-Samani M. Jamil A. Salvador R. Ruffini G. Haueisen J. Nitsche M. A. (2021). The impact of individual electrical fields and anatomical factors on the neurophysiological outcomes of tDCS: a TMS-MEP and MRI study. Brain Stimul. 14, 316–326. doi: 10.1016/j.brs.2021.01.016, PMID: 33516860

[ref57] Muthalib M. Kan B. Nosaka K. Perrey S. (2013). Effects of transcranial direct current stimulation of the motor cortex on prefrontal cortex activation during a neuromuscular fatigue task: an fNIRS study. Adv. Exp. Med. Biol. 789, 73–79. doi: 10.1007/978-1-4614-7411-1_11, PMID: 23852479

[ref58] Nitsche M. A. Paulus W. (2000). Excitability changes induced in the human motor cortex by weak transcranial direct current stimulation. J. Physiol. 527, 633–639. doi: 10.1111/j.1469-7793.2000.t01-1-00633.x, PMID: 10990547 PMC2270099

[ref59] Nitsche M. A. Seeber A. Frommann K. Klein C. C. Rochford C. Nitsche M. S.. (2005). Modulating parameters of excitability during and after transcranial direct current stimulation of the human motor cortex. J. Physiol. 568, 291–303. doi: 10.1113/jphysiol.2005.092429, PMID: 16002441 PMC1474757

[ref60] Opitz A. Paulus W. Will S. Antunes A. Thielscher A. (2015). Determinants of the electric field during transcranial direct current stimulation. NeuroImage 109, 140–150. doi: 10.1016/j.neuroimage.2015.01.033 25613437

[ref61] Pearson K. (2000). Motor systems. Curr. Opin. Neurobiol. 10, 649–654. doi: 10.1016/s0959-4388(00)00130-6 11084328

[ref62] Rawji V. Ciocca M. Zacharia A. Soares D. Truong D. Bikson M.. (2018). tDCS changes in motor excitability are specific to orientation of current flow. Brain Stimul. 11, 289–298. doi: 10.1016/j.brs.2017.11.001, PMID: 29146468 PMC5805821

[ref63] Rossini P. M. Burke D. Chen R. Cohen L. G. Daskalakis Z. Di Iorio R.. (2015). Non-invasive electrical and magnetic stimulation of the brain, spinal cord, roots and peripheral nerves: basic principles and procedures for routine clinical and research application. An updated report from an I.F.C.N Committee. Clin. Neurophysiol. 126, 1071–1107. doi: 10.1016/j.clinph.2015.02.001 25797650 PMC6350257

[ref64] Sriraman A. Oishi T. Madhavan S. (2014). Timing-dependent priming effects of tDCS on ankle motor skill learning. Brain Res. 1581, 23–29. doi: 10.1016/j.brainres.2014.07.021, PMID: 25063361 PMC4166556

[ref65] Tanaka S. Hanakawa T. Honda M. Watanabe K. (2009). Enhancement of pinch force in the lower leg by anodal transcranial direct current stimulation. Exp. Brain Res. 196, 459–465. doi: 10.1007/s00221-009-1863-9, PMID: 19479243 PMC2700246

[ref66] Tanaka S. Takeda K. Otaka Y. Kita K. Osu R. Honda M.. (2011). Single session of transcranial direct current stimulation transiently increases knee extensor force in patients with hemiparetic stroke. Neurorehabil. Neural Repair 25, 565–569. doi: 10.1177/1545968311402091, PMID: 21436391

[ref67] Tatemoto T. Yamaguchi T. Otaka Y. Kondo K. Tanaka S. (2013). Anodal transcranial direct current stimulation over the lower limb motor cortex increases the cortical excitability with Extracephalic reference electrodes. Conv. Clin. Eng. Res. Neurorehabil. 1:135. doi: 10.1007/978-3-642-34546-3_135

[ref68] Temesi J. Ly S. N. Millet G. Y. (2017). Reliability of single- and paired-pulse transcranial magnetic stimulation for the assessment of knee extensor muscle function. J. Neurol. Sci. 375, 442–449. doi: 10.1016/j.jns.2017.02.037, PMID: 28320184

[ref69] Truong D. Q. Magerowski G. Blackburn G. L. Bikson M. Alonso-Alonso M. (2013). Computational modeling of transcranial direct current stimulation (tDCS) in obesity: impact of head fat and dose guidelines. NeuroImage Clin. 2, 759–766. doi: 10.1016/j.nicl.2013.05.011, PMID: 24159560 PMC3778260

[ref70] van der Cruijsen J. Dooren R. F. Schouten A. C. Oostendorp T. F. Frens M. A. Ribbers G. M.. (2022). Addressing the inconsistent electric fields of tDCS by using patient-tailored configurations in chronic stroke: implications for treatment. NeuroImage Clin. 36:103178. doi: 10.1016/j.nicl.2022.103178, PMID: 36084558 PMC9465435

[ref71] Vargas V. Z. Baptista A. F. Pereira G. O. C. Pochini A. C. Ejnisman B. Santos M. B.. (2018). Modulation of isometric quadriceps strength in soccer Players with transcranial direct current stimulation: a crossover study. J. Strength Cond. Res. 32, 1336–1341. doi: 10.1519/JSC.0000000000001985, PMID: 28489629

[ref72] Vitor-Costa M. Okuno N. M. Bortolotti H. Bertollo M. Boggio P. S. Fregni F.. (2015). Improving cycling performance: transcranial direct current stimulation increases time to exhaustion in cycling. PLoS One 10:e0144916. doi: 10.1371/journal.pone.0144916, PMID: 26674200 PMC4687680

[ref73] Wang B. Xiao S. Yu C. Zhou J. Fu W. (2021). Effects of transcranial direct current stimulation combined with physical training on the excitability of the motor cortex, physical performance, and motor learning: a systematic review. Front. Neurosci. 15:648354. doi: 10.3389/fnins.2021.648354, PMID: 33897361 PMC8062775

[ref74] Washabaugh E. P. Santos L. Claflin E. S. Krishnan C. (2016). Low-level intermittent quadriceps activity during transcranial direct current stimulation facilitates knee extensor force-generating capacity. Neuroscience 329, 93–97. doi: 10.1016/j.neuroscience.2016.04.037, PMID: 27138643

[ref75] Wiethoff S. Hamada M. Rothwell J. C. (2014). Variability in response to transcranial direct current stimulation of the motor cortex. Brain Stimul. 7, 468–475. doi: 10.1016/j.brs.2014.02.003 24630848

[ref76] Yamaguchi T. Fujiwara T. Tsai Y. A. Tang S. C. Kawakami M. Mizuno K.. (2016). The effects of anodal transcranial direct current stimulation and patterned electrical stimulation on spinal inhibitory interneurons and motor function in patients with spinal cord injury. Exp. Brain Res. 234, 1469–1478. doi: 10.1007/s00221-016-4561-4, PMID: 26790423 PMC4851690

[ref77] Yeo B. T. Sabuncu M. R. Vercauteren T. Ayache N. Fischl B. Golland P. (2010). Spherical demons: fast diffeomorphic landmark-free surface registration. IEEE Trans. Med. Imaging 29, 650–668. doi: 10.1109/TMI.2009.2030797, PMID: 19709963 PMC2862393

[ref78] Yoon M. J. Park H. J. Yoo Y. J. Oh H. M. Im S. Kim T. W.. (2024). Electric field simulation and appropriate electrode positioning for optimized transcranial direct current stimulation of stroke patients: an in silico model. Sci. Rep. 14:2850. doi: 10.1038/s41598-024-52874-y 38310134 PMC10838316

