# Supplementary material for: Simulating tDCS electrode placement to stimulate both M1 and SMA enhances motor performance and modulates cortical excitability depending on current flow direction
Source: Front Neurosci. 2024 Jul 1;18:1362607. doi: 10.3389/fnins.2024.1362607 (PMC11246916; doi:10.3389/fnins.2024.1362607)
Supplement: Supplementary file 1 [file Data_Sheet_1.PDF]

Table S1. Effects of electrical conductivity on the electric field strength.

The average electric field strength over the four regions of interest was calculated when the conductivities of bone and CSF were altered from the default values. Data are presented as mean and ± standard deviation over 62 head models. The value in parentheses indicates the number of individual head models for which the electrode configuration was optimal.

		**Average electric field strength**				
		Default conductivity	Bone conductivity		CSF conductivity	
Electrode 1	Electrode 2		–50%	+50%	–10%	+10%
Fz	Extracephalic	0.36 ± 0.05 (0)	0.34 ± 0.05 (0)	0.37 ± 0.06 (0)	0.38 ± 0.05 (0)	0.35 ± 0.05 (0)
FCz	Extracephalic	0.49 ± 0.08 (0)	0.44 ± 0.07 (0)	0.52 ± 0.08 (0)	0.51 ± 0.08 (0)	0.47 ± 0.08 (0)
Cz	Extracephalic	0.57 ± 0.09 (0)	0.50 ± 0.08 (0)	0.61 ± 0.10 (0)	0.60 ± 0.09 (0)	0.55 ± 0.09 (0)
CPz	Extracephalic	0.53 ± 0.09 (0)	0.48 ± 0.08 (0)	0.57 ± 0.10 (0)	0.57 ± 0.09 (0)	0.52 ± 0.09 (0)
Pz	Extracephalic	0.44 ± 0.07 (0)	0.40 ± 0.07 (0)	0.47 ± 0.08 (0)	0.47 ± 0.07 (0)	0.43 ± 0.07 (0)
Fz	Iz	0.47 ± 0.06 (0)	0.43 ± 0.06 (0)	0.49 ± 0.07 (0)	0.49 ± 0.06 (0)	0.46 ± 0.06 (0)
FCz	Iz	0.56 ± 0.08 (0)	0.50 ± 0.07 (0)	0.60 ± 0.09 (0)	0.59 ± 0.08 (0)	0.55 ± 0.08 (0)
Cz	Iz	0.60 ± 0.09 (1)	0.52 ± 0.08 (2)	0.64 ± 0.10 (2)	0.63 ± 0.09 (1)	0.58 ± 0.09 (1)
CPz	Iz	0.50 ± 0.08 (0)	0.45 ± 0.08 (0)	0.54 ± 0.09 (0)	0.54 ± 0.09 (0)	0.49 ± 0.09 (0)
Pz	Iz	0.36 ± 0.06 (0)	0.32 ± 0.06 (0)	0.38 ± 0.07 (0)	0.38 ± 0.06 (0)	0.35 ± 0.06 (0)
Fz	Fpz	0.17 ± 0.04 (0)	0.16 ± 0.03 (0)	0.18 ± 0.04 (0)	0.18 ± 0.04 (0)	0.16 ± 0.04 (0)
FCz	Fpz	0.35 ± 0.07 (0)	0.31 ± 0.06 (0)	0.37 ± 0.08 (0)	0.37 ± 0.07 (0)	0.34 ± 0.07 (0)
Cz	Fpz	0.52 ± 0.09 (0)	0.46 ± 0.08 (0)	0.56 ± 0.10 (0)	0.55 ± 0.10 (0)	0.50 ± 0.10 (0)
CPz	Fpz	0.59 ± 0.09 (2)	0.52 ± 0.09 (3)	0.63 ± 0.10 (2)	0.62 ± 0.10 (2)	0.57 ± 0.10 (2)
Pz	Fpz	0.56 ± 0.08 (0)	0.51 ± 0.07 (0)	0.60 ± 0.08 (0)	0.60 ± 0.08 (0)	0.55 ± 0.08 (0)
POz	Fpz	0.49 ± 0.06 (0)	0.46 ± 0.06 (0)	0.51 ± 0.06 (0)	0.52 ± 0.06 (0)	0.48 ± 0.06 (0)
Fz	POz	0.61 ± 0.08 (0)	0.55 ± 0.07 (3)	0.64 ± 0.08 (0)	0.64 ± 0.08 (0)	0.59 ± 0.08 (0)
FCz	POz	0.65 ± 0.09 (32)	0.56 ± 0.08 (23)	0.70 ± 0.09 (29)	0.68 ± 0.09 (29)	0.63 ± 0.09 (33)
Cz	POz	0.59 ± 0.08 (1)	0.49 ± 0.07 (0)	0.64 ± 0.09 (1)	0.61 ± 0.08 (1)	0.57 ± 0.08 (1)
CPz	POz	0.37 ± 0.06 (0)	0.30 ± 0.05 (0)	0.41 ± 0.07 (0)	0.39 ± 0.06 (0)	0.36 ± 0.06 (0)
Fz	Pz	0.64 ± 0.09 (22)	0.57 ± 0.08 (31)	0.69 ± 0.10 (14)	0.68 ± 0.09 (25)	0.63 ± 0.09 (22)
FCz	Pz	0.63 ± 0.09 (4)	0.53 ± 0.08 (0)	0.69 ± 0.10 (14)	0.67 ± 0.09 (4)	0.62 ± 0.09 (3)
Cz	Pz	0.48 ± 0.07 (0)	0.37 ± 0.06 (0)	0.53 ± 0.08 (0)	0.50 ± 0.07 (0)	0.46 ± 0.07 (0)
